# Supplementary material for: Synthesis and Property Examination of Er2FeSbO7/BiTiSbO6 Heterojunction Composite Catalyst and Light-Catalyzed Retrogradation of Enrofloxacin in Pharmaceutical Waste Water under Visible Light Irradiation
Source: Materials (Basel). 2022 Aug 26;15(17):5906. doi: 10.3390/ma15175906 (PMC9457414; doi:10.3390/ma15175906)
Supplement: Supplementary file 1 [file materials-15-05906-s001.zip › materials-1560688-supplementary.pdf]

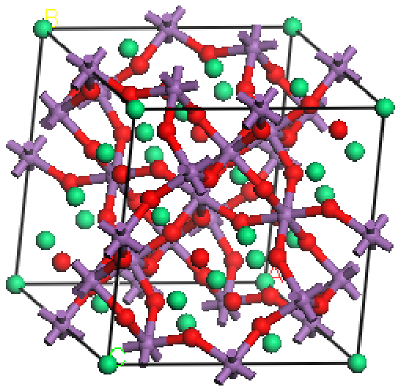

**Figure S1.** Atomic structure of $Er_2FeSbO_7$. (Red atom: O, green atom: Er, purple atom: Fe or Sb).

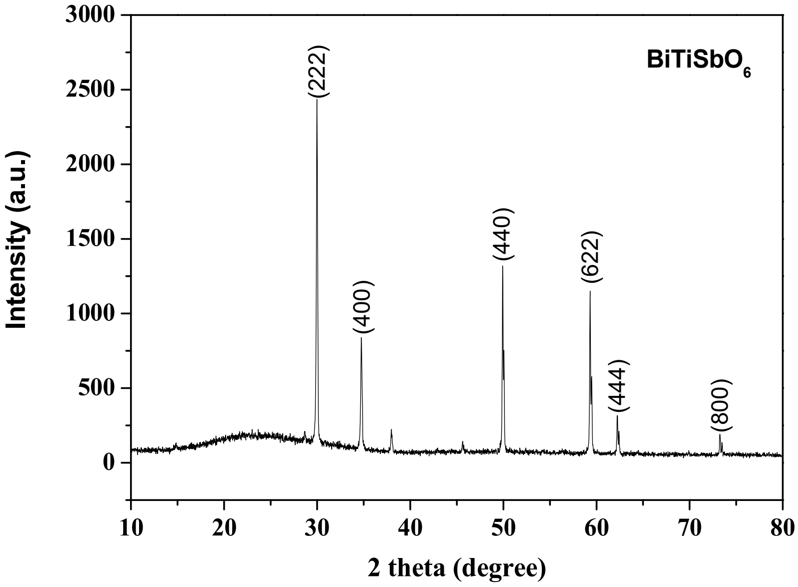


**Figure S2.** The X-ray diffraction spectrum of $BiTiSbO_6$ .

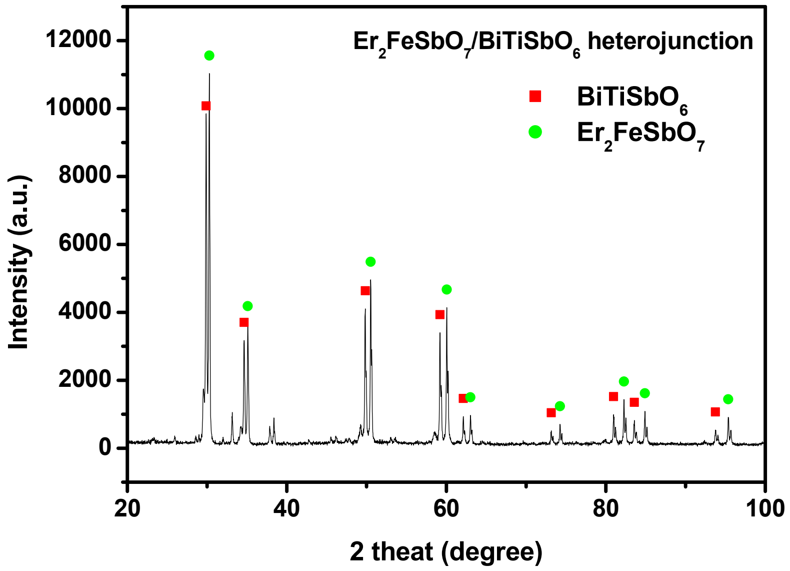


**Figure S3.** The X-ray diffraction pattern of $Er_2FeSbO_7/BiTiSbO_6$ heterojunction after the reaction.

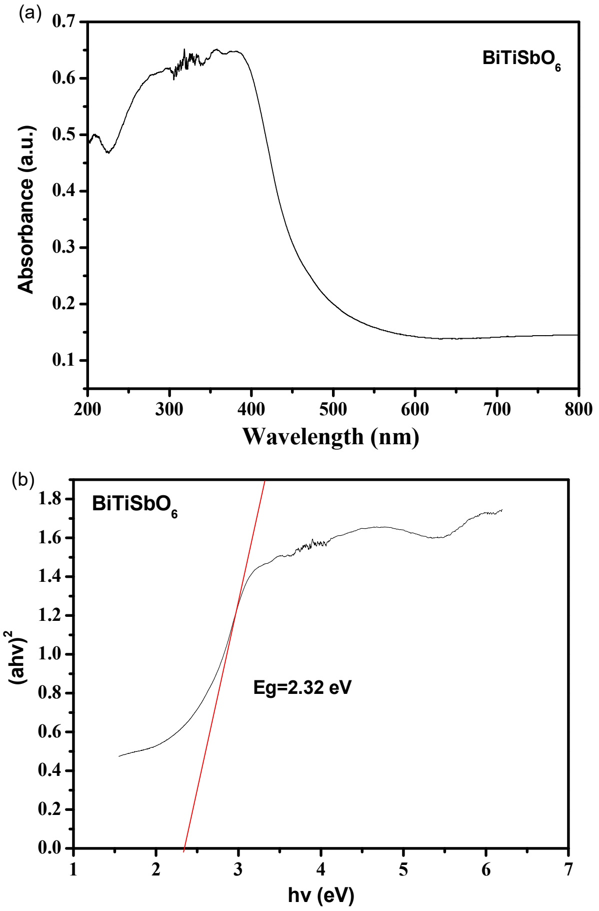


**Figure S4. (a)** UV-Vis diffuse reflectance spectra of $BiTiSbO_6$; **(b)** Plot of $(\alpha h\nu)^2$ versus $h\nu$ for $BiTiSbO_6$.

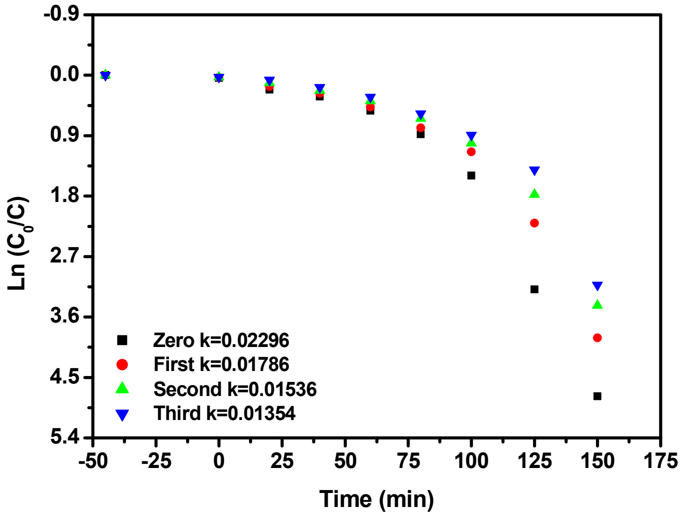


**Figure S5.** Observed first-order kinetic plots for the PCD of ENR with EBH as photocatalyst under VLGI for three cycle degradation tests.
